# Complete Genome Sequences of Eight Streptococcus equi subsp. *zooepidemicus* Strains Isolated from Mares in Estrus with Endometritis

**DOI:** 10.1128/MRA.01321-20

**Published:** 2021-07-01

**Authors:** Grace I. Borlee, Steven M. Lakin, Marylee L. Kapuscinski, Zaid Abdo, Mark D. Stenglein, Patrick M. McCue, Bradley R. Borlee

**Affiliations:** a Department of Microbiology, Immunology, and Pathology, Colorado State University, Fort Collins, Colorado, USA; b Department of Clinical Sciences, Colorado State University, Fort Collins, Colorado, USA; University of Maryland School of Medicine

## Abstract

Eight isolates of Streptococcus equi subsp. *zooepidemicus* were isolated from mares with clinical cases of endometritis. S. equi subsp. *zooepidemicus* strains were chosen for sequencing based on differing levels of biofilm production *in vitro*. Using Illumina short-read sequencing in conjunction with MinION sequencing, we report the genomes of eight isolates.

## ANNOUNCEMENT

Infectious endometritis, one of the leading causes of reduced fertility in mares, can be caused by fungal pathogens or bacterial pathogens such as Streptococcus equi subsp. *zooepidemicus*, Escherichia coli, Pseudomonas aeruginosa, and Klebsiella pneumoniae (1–4). A total of eight S. equi subsp. *zooepidemicus* isolates were chosen on the basis of their *in vitro* biofilm production using the microtiter dish biofilm formation assay (5). Three of the strains produced very little or no biofilm, two strains were medium biofilm producers, and three strains were high biofilm producers (Table 1).

**TABLE 1:** Characteristics and accession numbers of Streptococcus equi subsp. *zooepidemicus* genomes

Isolate	Age of mare (yr)	Biofilm production level	No. of Illumina reads	SRA accession no. for Illumina reads	No. of MinION reads	SRA accession no. for MinION reads	Plasmid size (bp)	GenBank accession no. for plasmid	Assembly size (bp)	No. of coding sequences	G+C content (%)	GenBank accession no.
SEZ13	13	Low	3,631,288	SRR12988984	15,448	SRR12995728	4,723	CP065055	2,150,055	1,950	41.6	CP065054
SEZ14	9	Low	2,598,319	SRR12988983	127,002	SRR12995727	4,652	CP065057	2,147,596	2,003	41.6	CP065056
SEZ18	10	Medium	2,183,355	SRR12988982	92,263	SRR12995726	NA^*a*^		2,082,352	1,890	41.6	CP065058
SEZ25	10	High	2,147,048	SRR12988981	80,784	SRR12995725	NA		2,098,084	1,949	41.6	CP065059
SEZ28	12	Low	1,988,827	SRR12988980	77,047	SRR12995724	NA		2,044,877	1,841	41.7	CP065060
SEZ33	4	High	1,577,737	SRR12988979	79,480	SRR12995723	NA		2,040,445	1,825	41.7	CP065061
SEZ36	10	High	3,550,098	SRR12988978	115,841	SRR12995722	NA		2,140,969	2,001	41.7	CP065190
SEZ46	14	Medium	2,350,585	SRR12988977	43,338	SRR12995721	NA		2,154,650	1,996	41.4	CP065191

a NA, not applicable.

S. equi subsp. *zooepidemicus* isolates were collected from the uterus of mares (age range, 4 to 13 years) in estrus using a sterile, double-guarded uterine swab during the breeding season of March 2017 to May 2018. Isolates of S. equi subsp. *zooepidemicus* were streaked for purity on tryptic soy agar (TSA) with 5% sheep blood plates. A single colony from each sample was grown overnight in Todd-Hewitt broth at 37°C in 5% CO_2_. Cryopreserved isolates were used for identification using matrix-assisted laser desorption ionization–time of flight mass spectrometry (MALDI-TOF MS) (6). S. equi subsp. *zooepidemicus* isolates were grown statically overnight in 2 ml Todd-Hewitt broth with 0.5% Tween 80 at 37°C in 5% CO_2_. Genomic DNA was isolated from the bacterial cell pellet using the Qiagen DNeasy blood and tissue kit, following the manufacturer’s instructions for Gram-positive bacteria.

For DNA sequencing and bioinformatic processing, a hybrid assembly approach utilized Illumina short reads and Oxford Nanopore Technologies (ONT) long reads obtained by MinION sequencing, which produced a single contig per genome or plasmid. Genomic DNA was sequenced on an Illumina NextSeq 550 sequencer from a 2 × 150-bp paired-end library that was prepared at the Microbial Genome Sequencing Center (MiGS) (University of Pittsburgh) using the Nextera DNA library preparation kit with modifications that included the substitution of KAPA HiFi PCR mix and a modified PCR protocol that used 7 cycles to attach indices followed by an additional 5 cycles after the addition of P5/P7 Illumina primers, as described by Baym et al. (7). Long-read sequencing was performed at Colorado State University using the ONT MinION platform with an R9 MinION flow cell using the rapid barcoding kit (P/N SQK-RBK004). The DNA was fragmented during tagmentation but was not size selected. The ONT read *N*_50_ value (total for all data sets) was 6,618 bp. Base calling was performed using Guppy (v3.3.0). FastQ files from Illumina sequencing were assessed for quality using FastQC (v0.11.8) (8) and were trimmed using Cutadapt (v2.5) (9) to remove sequences that were less than 80 bp and those that were below a minimum quality score of 30 on the Phred scale. MinION reads and trimmed Illumina reads were assembled using the SPAdes hybrid assembler (v3.13.1) (10) and Unicycler (v0.4.8) (11). S. equi subsp. *zooepidemicus* genomes were annotated with Prokka (v1.14.0) (12). Prokka annotations for all genomes can be found in Table 1. To determine the start position within each genome, *dnaA* from the S. equi subsp. *zooepidemicus* H70 genome (GenBank accession number FM204884.1) (13) was aligned to the chromosomal assemblies using the Burrows-Wheeler Aligner (v0.7.17) (14). Using an in-house Python script (https://github.com/lakinsm/strep-equi-mra), the chromosomal assemblies were aligned, reverse complemented, and circularly shifted so that all chromosomal assemblies had the same orientation and start position. Visualization and additional analyses were performed using Geneious Prime (v2019.2.1). Default parameters were used except where otherwise noted.

S. equi subsp. *zooepidemicus* genomes ranged from 2,040,445 to 2,154,650 bp (Table 1). Two of the strains, SEZ13 and SEZ14 (both low biofilm producers), maintained plasmids of 4,723 bp and 4,652 bp, respectively.

### Data availability.

All eight S. equi subsp. *zooepidemicus* genomes are associated with BioProject PRJNA673199. Illumina and MinION raw reads are available under the accession numbers listed in Table 1.
